# Supplementary material for: Conditional chemoconnectomics (cCCTomics) as a strategy for efficient and conditional targeting of chemical transmission
Source: eLife. 2024 Apr 30;12:RP91927. doi: 10.7554/eLife.91927 (PMC11060718; doi:10.7554/eLife.91927)
Supplement: Figure 3—source data 3. [file elife-91927-fig3-data3.zip › Figure 3 source data3 part 2.zip!/Figure 3-Source data 3-part2/1063-1-CRISPResso_Report_az97gqtx.zip!/CRISPResso_on_1063_4/2a.Nucleotide_percentage_quilt.pdf]

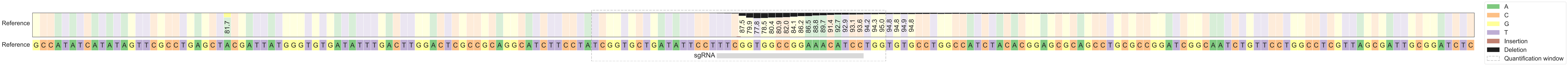

Reference
81.7
87.5
79.1
77.8
78.5
80.4
80.9
82.0
84.1
86.1
86.5
88.8
89.7
91.4
92.7
92.9
93.1
93.6
94.2
94.3
95.0
94.8
94.8
94.8
94.9
94.8
Reference
GCCATATCATATAGTTCGCCTGAGCTACGATTATGGGTGTGATATTTGACTTGGACTCGCCGCAGGCATCTTCCTATCGGTGCTGATATTCCTTTCGGTGGCCGGAAACATCCTGGTGTGCCTGGCCATCTACACGGAGCGCAGCCTGCGCCGGATCGGCAATCTGTTCCTGGCCTCGTTAGCGATTGCGGATCTC
sgRNA
A
C
G
T
Insertion
Deletion
Quantification window
